# Prevalence of intimate partner violence and abuse and associated factors among women enrolled into a cluster randomised trial in northwestern Tanzania

**DOI:** 10.1186/s12889-017-4119-9

**Published:** 2017-02-14

**Authors:** Saidi Kapiga, Sheila Harvey, Abdul Khalie Muhammad, Heidi Stöckl, Gerry Mshana, Ramadhan Hashim, Christian Hansen, Shelley Lees, Charlotte Watts

**Affiliations:** 1 Mwanza Intervention Trials Unit, PO Box 11936, Mwanza, Tanzania; 2 Department of Infectious Diseases Epidemiology, London School of Hygiene & Tropical Medicine, Keppel Street, London, WC1E 7HT UK; 3 Department of Global Health and Development, London School of Hygiene & Tropical Medicine, Keppel Street, London, WC1E 7HT UK; 4 National Institute for Medical Research, Isamilo Road, Mwanza, Tanzania

**Keywords:** Intimate partner violence, Economic abuse, Emotional abuse, Controlling behaviour, Mental health, Intervention, Cross-sectional study, Women, Tanzania, Africa

## Abstract

**Background:**

Intimate partner violence (IPV) is recognised as an important public health and social problem, with far reaching consequences for women’s physical and emotional health and social well-being. Furthermore, controlling behaviour by a partner has a similar impact on women’s well-being, yet little is known about the prevalence of this type of behaviour and other related abuses in Tanzania and in other sub-Saharan African countries.

**Methods:**

We conducted a cross-sectional study to determine the lifetime and past 12-month prevalence of physical and sexual IPV, economic abuse, emotional abuse and controlling behaviour among ever-partnered women in Mwanza, Tanzania. Women (*N* = 1049) were enrolled in an ongoing trial (Maisha study) to assess the impact of microfinance combined with gender training on participants’ experience IPV, and other related outcomes. Interviews were conducted by same sex interviewers to collect information about socio-demographic characteristics, experiences of specific acts of IPV and abuse, and symptoms of poor mental health status.

**Results:**

Overall, about 61% of women reported ever experiencing physical and/or sexual IPV (95% CI: 58–64%) and 27% (95% CI: 24–29%) experienced it in the past 12 months. Partner controlling behaviour was the most prevalent type of abuse with 82% experiencing it in their lifetime and 63% during the past 12 months. Other types of abuses were also common, with 34% of women reporting economic abuse and 39% reporting emotional abuse during the past 12 months. The prevalence of IPV and abuses varied by socio-demographic characteristics, showing much higher prevalence rates among younger women, women with young partners and less educated women. After we adjusted for age and socio-economic status, physical violence (OR = 1.8; 95% CI: 1.3–2.7) and sexual violence (OR = 2.8; 95% CI: 1.9–4.1) were associated with increased reporting of symptoms of poor mental health. Similarly, experience of abuse during the past 12 months was associated with increased reporting of symptoms of poor mental health.

**Conclusions:**

The high prevalence of IPV and abuses and its strong links with symptoms of poor mental health underline the urgent need for developing and testing appropriate interventions in settings like Tanzania to tackle both violence and abusive behaviours among intimate partners.

**Trial registration:**

ClinicalTrials.gov – ID NCT02592252, registered retrospectively on 13 August 2015.

## Background

Intimate partner violence (IPV) is recognised as an important public health problem, development issue and human rights concern. Globally, it is estimated that about 30% of women will experience physical and/or sexual violence from an intimate partner during their lifetime [1], and that one in three homicides among women are by an intimate partner [2]. Lifetime rates of physical and/or sexual IPV have been found to be highest in south-East Asia, the Mediterranean region and sub-Saharan Africa [1]. In Tanzania, almost 2 in 5 women aged 15 to 49 years have experienced physical violence at some point in their lives, 44% of ever-married women have experienced physical and/or sexual violence by their current or most recent husband or partner, and 37% of ever-married women experienced such spousal violence in the past 12 months [3]. Results from the baseline survey of an ongoing cluster-randomized HIV and gender-based violence prevention trial in Dar es Salaam, Tanzania showed that within the last 12 months, 35.8% of women reported experiencing physical, sexual and/or psychological IPV [4].

Experience of IPV has far reaching consequences for women’s physical and emotional health and social well-being [5–7]. Women who experience IPV show more physical symptoms of poor health, and more days out of work and injuries than women who have not been abused [5, 7]. IPV has also been associated with mental health problems, including depression, anxiety, phobias, post-traumatic stress disorder, suicide, and alcohol and drug abuse [6–10]. Furthermore, recent evidence suggests that controlling behaviour by a partner has a similar impact on women’s well-being [11], yet little is known about the prevalence of this type of behaviour and other related abuses in Tanzania.

Several intervention studies have been implemented in low and middle income countries to address IPV [12–15]. One example is the Intervention with Microfinance for AIDS and Gender Equity (IMAGE) trial implemented in rural South Africa [13], which found that a gender empowerment intervention linked to microfinance loans was associated with 55% reduced IPV incidence in the past year. We are currently conducting a trial (MAISHA study), to adapt and evaluate the impact of the IMAGE model in Mwanza city, northwestern Tanzania.

In this paper, we describe the design of the MAISHA study and present the baseline lifetime and past 12-month prevalence and severity of physical and sexual IPV and other abuses, including economic abuse, emotional abuse and controlling behaviour. Additionally, we assessed the co-occurrence (or overlap) of different forms of violence and abuses, and the associations between experience of violence and abuses, and socio-demographic characteristics and women’s self-reported mental health status.

## Methods

### MAISHA study design and procedures

MAISHA study is a cluster-randomised controlled trial to assess the impact of a combined microfinance and gender training programme for women on participants’ experience of physical and/or sexual violence, as well as other gender-empowerment, economic, and health related outcomes. The trial is a collaboration with an established local microfinance provider in Tanzania (Bangladesh Rural Advancement Committee or BRAC). As part of BRAC microfinance programme standard procedures, loans are provided to women in groups, with about 10–30 women per group. We recruited into the trial 66 existing BRAC microfinance loan groups from low socio-economic communities in Mwanza city. Each woman in the groups approached for this study met with a staff member to go through the participant information sheet and those who agreed to participate and demonstrated understanding of the study procedures were invited to sign the consent form. Following completion of baseline data collection, loan groups were randomly allocated in a 1:1 ratio to either continue with microfinance only (*N* = 33 groups) or to receive a combined microfinance and gender training intervention (*N* = 33 groups). The gender training intervention included 10 sessions delivered by female facilitators over 20 weeks at a venue convenient to the participants. The training was developed drawing on six curricula including the Sisters For Life curriculum originally developed for the IMAGE trial [13].

### Baseline survey

During the baseline survey, consenting women were interviewed, face-to-face, using a structured questionnaire, with responses entered directly onto a tablet computer programmed to check for accuracy and consistency of information entered during the interview. Interviews were conducted in a private location by female interviewers trained on interviewing techniques, gender issues, violence, and ethical issues related to research on IPV. We used a standardised structured questionnaire which was translated into a local language (Swahili) and then independently back-translated into English to check for accuracy. The questionnaire included information about household details, income, relationships, health, childhood and experiences of specific acts of IPV and abuse. Symptoms of poor mental health were assessed using a standardised tool (SRQ-20), integrated into the health section of the questionnaire, which was developed by WHO [16] and has been validated in a range of populations [17]. Questions asking about each type of violence and abuse were adapted from the WHO Violence Against Women instrument (Table 1) [18].

**Table 1:** Questions used in the study to document violence, controlling behaviours, emotional abuse and economic abuse by an intimate partner in Mwanza, Tanzania

	Questions
Physical violence	Has your current partner or any other partner ever…
	1. Slapped you or thrown something at you that could hurt you?
	2. Pushed you or shoved you or pulled your hair?
	3. Hit you with his fist or with something else that could hurt you?
	4. Kicked you, dragged you or beaten you up?
	5. Choked or burnt you on purpose?
	6. Threatened to use or actually used a gun, knife or other weapon against you?
Sexual violence	Have you ever had sexual intercourse with your current partner or other partner
	1. After he forced you by threatening you, holding you down or hurting you in some way?
	2. When you did not want to because you were afraid that your partner would hurt you or someone you cared about if you refused?
	3. When you did not want to because you were afraid that your partner would leave you or take another girlfriend if you refused?
Controlling behaviours	Thinking about your (current or most recent or past) partner, would you say it is generally true that he:
	1. Tries to keep you from seeing your friends
	2. Tries to restrict contact with your family of birth
	3. Insists on knowing where you are at all times
	4. Is jealous and gets angry if you speak with another man
	5. Is often suspicious that you are unfaithful
Economic abuse	Thinking about your (current or most recent/past) partner, would you say it is generally true that he:
	1. Refuses to give you enough money for household expenses, even when he has money for other things?
	2. Takes money that you have earned away from you
	3. Makes important financial decisions without consulting you
Emotional abuse	Has your current partner, or any other partner ever…
	1. Insulted you or made you feel bad about yourself?
	2. Belittled or humiliated you in front of other people?
	3. Done things to scare or intimidate you on purpose (e.g. by the way he looked at you, by yelling and smashing things)?
	4. Verbally threatened to hurt you or someone you care about?

In order to ensure support for participants who reported acts of violence or abuse perpetrated against them, we identified service providers in the communities where we recruited study participants. Our study team met with representatives of each service provider to inform them about the study and to establish effective mechanisms for referring women for ongoing help and support.

### Data analysis

Intimate partner violence was defined as one or more acts of physical and/or sexual violence and abuse was defined as one or more acts of either controlling behaviour, emotional abuse and/or economic abuse (Table 1). We defined the prevalence of ever experiencing IPV as the proportion of ever-partnered women (i.e. women who had ever been in a relationship including those who had ever been married or lived with a partner) reporting one or more acts of physical and/or sexual violence by an intimate partner at any point in their lives. Current prevalence of IPV was defined as the proportion of ever-partnered women reporting one or more acts of violence during the 12 months before the interview. The same approach was used to define prevalence of the different forms of abuse – controlling behaviour, emotional abuse and economic abuse.

Physical violence was considered severe if a participant reported having been hit, kicked, chocked or threatened with a weapon; and less severe if they reported having been pushed or slapped. For emotional abuse, economic abuse and controlling behaviours, severity was defined by the number of yes responses to the component questions of each abuse experienced by participant (Table 1).

We summarised the prevalence (with corresponding 95% confidence intervals (CI)) of each type of violent and abusive act experienced, overall and by severity. We also summarised the prevalence of current violent and abusive acts for each stratum of the following socio-demographic variables: women’s age, education, marital status, number of children, monthly earnings and overall socio-economic status; and male partner’s age and education. We used structural equation modelling [19] to generate a summary socio-economic status indicator from 19 variables collected in the questionnaire. Symptoms of poor mental health were assessed by using SRQ-20 cut-off point of 7/8 (yes to 8 or more questions is a case, 7 or less is a non-case). The associations between symptoms of poor mental health and different types of violence and abuses were explored by cross tabulations and logistic regression models. We summarised the associations firstly in terms of crude odds ratios and corresponding 95% CIs, and secondly after controlling for age, socio-economic status, violence and abuses. Analyses were done using Stata version 13.1 software (StataCorp, College Station, TX, USA).

## Results

Between September 2014 and June 2015, 66 out of 110 microfinance loan groups approached were recruited into the trial. Of the 1154 women in the 66 groups who received information about the trial, 1049 provided consent to join the study. Baseline interviews were conducted among 1021 women who were available at the time of the survey and reported to have ever been in a relationship. Women’s age ranged from 19 to 70 years, with a median age of 39 years (interquartile range [IQR] 33–46), and 28.7% were less than 35 years of age. Most participants were married or living with a man as if married (72.8%), and had completed primary education (72.3%).

### Prevalence of violence and different forms of abuses

Table 2 presents the lifetime and current prevalence of each type of violent and abusive act. Overall, about 61% of women reported ever experiencing physical and/or sexual IPV (95% CI: 58–64%) and 27% (95% CI: 24–29%) experienced it in the past 12 months. When examined separately, the prevalence of lifetime physical and sexual violence was 53 and 35%, respectively. The prevalence of physical and sexual violence during the past 12 months before the interviews was 19 and 17%, respectively. Overall, most women who had experienced physical violence in their lifetime (68%) or during the past 12 months (68%) reported severe forms of violence.

**Table 2:** Prevalence and severity of self-reported experiences of intimate partner violence and abuse among ever-partnered women (*N* = 1021) in Mwanza, Tanzania

	Lifetime experience				Experienced during the 12 months before interview			
	*N*	Percent	95% CI		*N*	Percent	95% CI	
Physical and/or sexual violence	621	60.8	57.8	63.8	271	26.5	23.9	29.4
Physical violence								
Overall^a^a	541	53.0	49.9	56.1	194	19.0	16.6	21.5
Less severe^c^	175	17.1	14.9	19.6	63	6.2	4.8	7.8
Severe^b^	366	35.8	32.9	38.9	131	12.8	10.8	15.0
Sexual violence								
Overall^a^	355	34.8	31.8	37.8	171	16.7	14.5	19.2
Controlling behaviour								
Overall^a^	832	81.5	79.0	83.8	639	62.6	59.5	65.6
Yes to only 1 question	256	25.1	22.4	27.9	250	24.5	21.9	27.2
Yes to ≥ 2 questions	576	56.4	53.3	59.5	389	38.1	35.1	41.2
Emotional abuse								
Overall^a^	694	68.0	65.0	70.8	401	39.3	36.3	42.3
Yes to only 1 question	267	26.2	23.5	29.0	184	18.0	15.7	20.5
Yes to ≥ 2 questions	427	41.8	38.8	44.9	217	21.3	18.8	23.9
Economic abuse								
Overall^a^	479	46.9	43.8	50.0	343	33.6	30.7	36.6
Yes to only 1 question	206	20.2	17.8	22.8	167	16.4	14.1	18.8
Yes to ≥ 2 questions	273	26.7	24.0	29.6	176	17.2	15.0	19.7

^a^For each type of violence/abuse, the overall prevalence was defined as the % of women who answered ‘yes’ to at least one question listed in Table 1. ^b^Women were classified as having experienced severe physical violence if they answered ‘yes’ to any of the last four physical violence questions listed in Table 1 (i.e. questions 3 to 6); and ^c^less severe if otherwise

Among other abuses, partner controlling behaviour was the most prevalent type of abuse with 82% experiencing it in their lifetime and 63% during the past 12 months. Other types of abuses were also common, with 34% of women reporting economic abuse and 39% reporting emotional abuse during the past 12 months. Most women who reported economic (51%) and emotional (54%) abuse during the past 12 months had responded ‘yes’ to at least two different acts of abuse.

### Co-occurrence of past 12-month violence and abuse

**Fig. 1:**
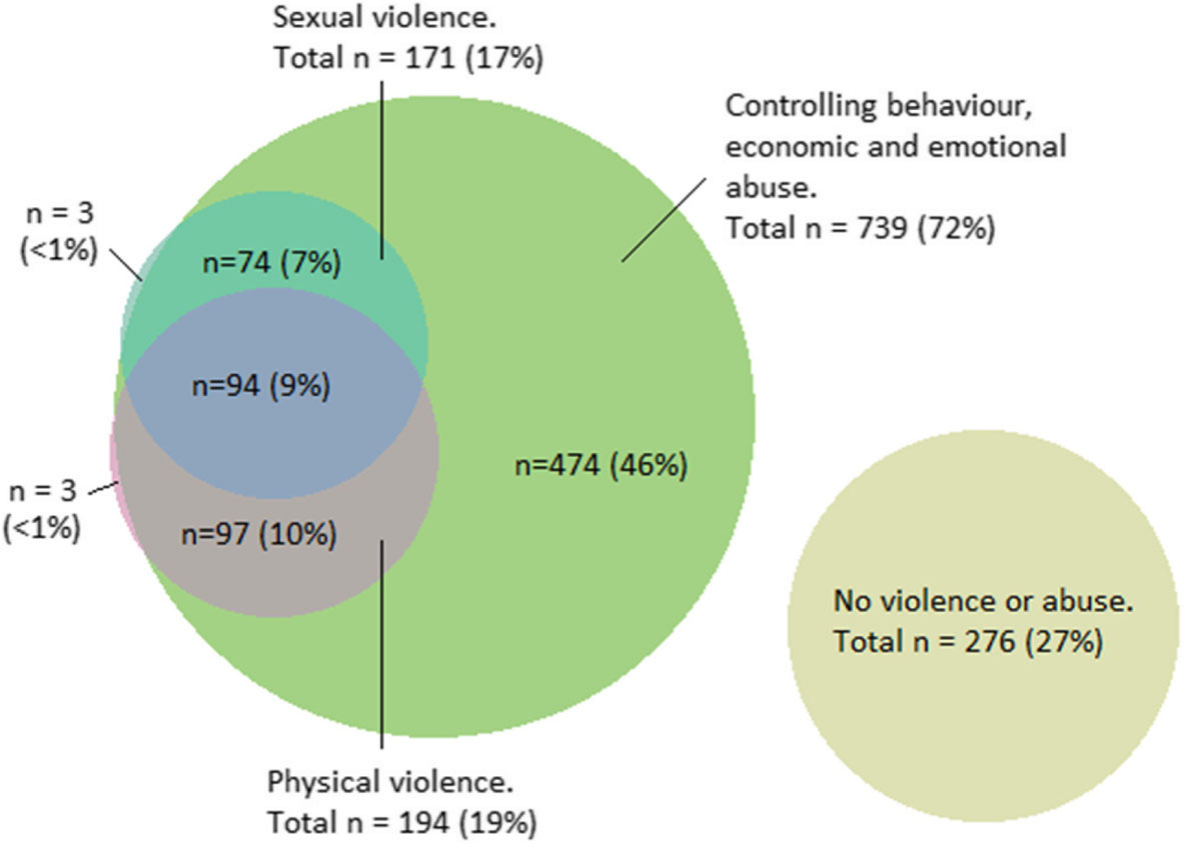
Co-occurrence of self-reported experience of violence and abuse in the past 12 months (*n* = 1021)

In Fig. 1, we present the co-occurrence (or overlaps) of reported violence and abuse during the past 12 months. Overall, there was considerable overlap between physical and sexual violence and the different forms of abuses, with only 276 (27%) women not reporting any violence or abuse during the past 12 months. Among 271 women who reported physical and/or sexual violence by their current partners in the last 12 months, 94 (35%) women experienced both physical and sexual violence; and almost all (98%) report at least one form of abuse within the same time period. Out of 739 women who experienced at least one form of abuse, 200 (27.1%) experienced all abuses (i.e. controlling behaviour, economic abuse and emotional abuse) in the last 12 months.

### Associations with socio-demographic characteristics

In Tables 3 and 4 we present the associations between experience of violence and different forms of abuse in the past 12 months and selected socio-demographic characteristics. The prevalence of physical and/or sexual violence was highest among women below 30 years of age, and among those with primary education or less. Overall, the prevalence of violence decreased with increasing women’s age and level of education. Similarly, the prevalence of violence decreased with increasing male partner’s age and level of education. For controlling behaviour, higher prevalence rates were also found among younger women. Economic abuse showed no distinct patterns, although the prevalence of economic and emotional abuses was relatively low among women ≥ 50 years and among those with post-secondary education. Women who were widowed and those without children below the age of 18 years were relatively less likely to experience violence or abuses when compared with other women. Women’s monthly earnings and socio-economic status was not related to experience of violence or abuses.

**Table 3:** The associations between experience of intimate partner violence during the past 12 months and socio-demographic characteristics among ever-partnered women in Mwanza, Tanzania

		Physical and/or sexual violence			Physical violence			Sexual violence		
	*N*	Percent	Exact 95% CI		Percent	Exact 95% CI		Percent	Exact 95% CI	
Age										
Less than 30 yrs	143	42.0	33.8	50.5	31.5	24.0	39.8	25.2	18.3	33.1
30–34 yrs	150	35.3	27.7	43.5	28.0	21.0	35.9	22.7	16.2	30.2
35–39 yrs	234	28.2	22.5	34.4	20.9	15.9	26.7	17.9	13.3	23.5
40–49 yrs	331	23.3	18.8	28.2	15.1	11.4	19.4	14.8	11.2	19.1
50+ yrs	163	9.2	5.2	14.7	4.9	2.1	9.4	6.1	3.0	11.0
Level of education										
No formal education	59	27.1	16.4	40.3	18.6	9.7	30.9	16.9	8.4	29.0
Primary	738	27.9	24.7	31.3	20.5	17.6	23.6	17.5	14.8	20.4
Secondary	200	23.0	17.4	29.5	15.5	10.8	21.3	14.5	9.9	20.2
Post-secondary education	24	12.5	2.7	32.4	4.2	0.1	21.1	12.5	2.7	32.4
Marital status										
Married/living with man as if married	743	30.6	27.3	34.0	22.6	19.6	25.8	19.2	16.5	22.3
Divorced/separated	151	17.9	12.1	24.9	13.2	8.3	19.7	10.6	6.2	16.6
Widowed	100	9.0	4.2	16.4	2.0	0.2	7.0	8.0	3.5	15.2
Never married	27	29.6	13.8	50.2	14.8	4.2	33.7	14.8	4.2	33.7
Monthly earnings^a^										
No personal earnings	31	22.6	9.6	41.1	9.7	2.0	25.8	16.1	5.5	33.7
Less than USD 60	247	33.2	27.4	39.4	26.3	20.9	32.3	20.2	15.4	25.8
USD 60 to 179	303	26.1	21.2	31.4	18.8	14.6	23.7	16.5	12.5	21.2
USD 180 to 499	165	17.6	12.1	24.3	12.1	7.6	18.1	12.1	7.6	18.1
above USD 500	252	25.8	20.5	31.7	17.9	13.3	23.2	14.7	10.6	19.7
Don’t know earnings	23	39.1	19.7	61.5	17.4	5.0	38.8	39.1	19.7	61.5
Partner’s age										
Less than 30 yrs	36	33.3	18.6	51.0	25.0	12.1	42.2	19.4	8.2	36.0
30–34 yrs	80	47.5	36.2	59.0	38.8	28.1	50.3	27.5	18.1	38.6
35–39 yrs	148	35.8	28.1	44.1	25.7	18.9	33.5	25.0	18.3	32.8
40–49 yrs	343	28.0	23.3	33.1	20.4	16.3	25.1	15.7	12.1	20.0
50+ yrs	329	17.3	13.4	21.9	10.6	7.5	14.5	12.5	9.1	16.5
Don’t know	85	17.6	10.2	27.4	12.9	6.6	22.0	11.8	5.8	20.6
Partner’s education										
No formal education	16	43.8	19.8	70.1	31.3	11.0	58.7	25.0	7.3	52.4
Primary	567	29.5	25.7	33.4	22.4	19.0	26.1	18.9	15.7	22.3
Secondary	316	24.1	19.4	29.2	15.8	12.0	20.3	14.6	10.9	18.9
Post-secondary education	85	20.0	12.1	30.1	11.8	5.8	20.6	12.9	6.6	22.0
Don’t know	37	10.8	3.0	25.4	5.4	0.7	18.2	8.1	1.7	21.9
Respondent’s number of children under 18 years										
No children	73	16.4	8.8	27.0	5.5	1.5	13.4	13.7	6.8	23.8
1 Child	151	23.2	16.7	30.7	12.6	7.7	19.0	15.2	9.9	22.0
2 Children	192	25.5	19.5	32.3	19.8	14.4	26.1	15.1	10.4	21.0
3 Children	225	31.6	25.5	38.1	23.1	17.8	29.2	20.9	15.8	26.8
4 Children	170	25.9	19.5	33.1	18.8	13.2	25.5	14.1	9.3	20.3
5 or more children	210	28.6	22.6	35.2	23.3	17.8	29.6	18.1	13.1	24.0
Socio-economic status quintile^b^										
1^st^	192	29.7	23.3	36.7	21.9	16.2	28.4	20.3	14.9	26.7
2^nd^	174	32.8	25.8	40.3	24.1	18.0	31.2	21.8	15.9	28.7
3^rd^	209	19.6	14.5	25.7	12.9	8.7	18.2	13.9	9.5	19.3
4^th^	198	26.8	20.7	33.5	20.7	15.3	27.0	13.1	8.8	18.6
5^th^	194	24.2	18.4	30.9	18.0	12.9	24.2	12.9	8.5	18.4
Unknown	54	29.6	18.0	43.6	13.0	5.4	24.9	25.9	15.0	39.7

^a^Participants reported their earnings in Tanzanian shillings in terms of either daily, weekly or monthly income. Reported daily and weekly earnings were converted to monthly earnings on the assumption that each participant worked for 22 days per month. The conversion to US Dollars (USD) was based on the exchange rate at the time of data collection (1USD = 1,887 Tanzanian shillings). ^b^A higher quintile indicates higher socio-economic status. The socio-economic status indicator was derived as a latent variable from 19 indicators collected in the questionnaire including education, earnings and household ownership of car, fridge, television and motorcycle

**Table 4:** The associations between experience of intimate partner abuse during the past 12 months and socio-demographic characteristics among ever-partnered women in Mwanza, Tanzania

		Controlling behaviour			Emotional abuse			Economic abuse		
	*N*	Percent	Exact 95% CI		Percent	Exact 95% CI		Percent	Exact 95% CI	
Age										
Less than 30 yrs	143	76.9	69.1	83.6	48.3	39.8	56.8	33.6	25.9	41.9
30–34 yrs	150	74.7	66.9	81.4	55.3	47.0	63.4	40.0	32.1	48.3
35–39 yrs	234	67.5	61.1	73.5	40.2	33.8	46.8	35.9	29.8	42.4
40–49 yrs	331	58.9	53.4	64.3	38.4	33.1	43.8	36.6	31.4	42.0
50+ yrs	163	39.3	31.7	47.2	17.2	11.7	23.9	18.4	12.8	25.2
Level of education										
No formal education	59	61.0	47.4	73.5	39.0	26.5	52.6	37.3	25.0	50.9
Primary	738	60.8	57.2	64.4	40.7	37.1	44.3	34.6	31.1	38.1
Secondary	200	69.5	62.6	75.8	36.0	29.4	43.1	30.0	23.7	36.9
Post-secondary education	24	62.5	40.6	81.2	25.0	9.8	46.7	25.0	9.8	46.7
Marital status										
Married/living with man as if married	743	70.5	67.1	73.8	46.3	42.7	50.0	36.6	33.1	40.2
Divorced/separated	151	47.7	39.5	56.0	24.5	17.9	32.2	28.5	21.4	36.4
Widowed	100	26.0	17.7	35.7	11.0	5.6	18.8	19.0	11.8	28.1
Never married	27	63.0	42.4	80.6	33.3	16.5	54.0	33.3	16.5	54.0
Monthly earnings^a^										
No personal earnings	31	77.4	58.9	90.4	48.4	30.2	66.9	35.5	19.2	54.6
Less than USD 60	247	59.1	52.7	65.3	44.1	37.8	50.6	35.6	29.7	41.9
USD 60 to 179	303	58.7	53.0	64.3	36.6	31.2	42.3	37.3	31.8	43.0
USD 180 to 499	165	63.6	55.8	71.0	37.6	30.2	45.4	26.1	19.5	33.5
above USD 500	252	66.3	60.1	72.1	36.9	30.9	43.2	30.2	24.6	36.2
Don’t know earnings	23	82.6	61.2	95.0	47.8	26.8	69.4	52.2	30.6	73.2
Partner’s age										
Less than 30 yrs	36	66.7	49.0	81.4	47.2	30.4	64.5	22.2	10.1	39.2
30–34 yrs	80	80.0	69.6	88.1	51.2	39.8	62.6	26.3	17.0	37.3
35–39 yrs	148	75.7	67.9	82.3	48.0	39.7	56.3	39.2	31.3	47.5
40–49 yrs	343	65.3	60.0	70.3	42.3	37.0	47.7	38.2	33.0	43.6
50+ yrs	329	55.0	49.5	60.5	31.6	26.6	36.9	30.1	25.2	35.4
Don’t know	85	40.0	29.5	51.2	27.1	18.0	37.8	30.6	21.0	41.5
Partner’s education										
No formal education	16	56.3	29.9	80.2	43.8	19.8	70.1	50.0	24.7	75.3
Primary	567	60.7	56.5	64.7	41.6	37.5	45.8	33.3	29.5	37.4
Secondary	316	65.5	60.0	70.7	39.6	34.1	45.2	34.8	29.6	40.3
Post-secondary education	85	70.6	59.7	80.0	30.6	21.0	41.5	28.2	19.0	39.0
Don’t know	37	51.4	34.4	68.1	18.9	8.0	35.2	32.4	18.0	49.8
Respondent's number of children under 18 years										
No children	73	47.9	36.1	60.0	28.8	18.8	40.6	27.4	17.6	39.1
1 Child	151	57.6	49.3	65.6	34.4	26.9	42.6	29.1	22.0	37.1
2 Children	192	62.0	54.7	68.9	39.1	32.1	46.3	33.3	26.7	40.5
3 Children	225	64.4	57.8	70.7	36.4	30.2	43.1	37.3	31.0	44.0
4 Children	170	63.5	55.8	70.8	43.5	36.0	51.3	34.1	27.0	41.8
5 or more children	210	69.0	62.3	75.2	46.2	39.3	53.2	34.8	28.3	41.6
Socio-economic status quintile^b^										
1^st^	192	57.3	50.0	64.4	42.2	35.1	49.5	29.7	23.3	36.7
2^nd^	174	63.2	55.6	70.4	44.8	37.3	52.5	33.9	26.9	41.5
3^rd^	209	64.1	57.2	70.6	34.9	28.5	41.8	33.5	27.1	40.3
4th	198	62.1	55.0	68.9	37.4	30.6	44.5	31.8	25.4	38.8
5th	194	61.3	54.1	68.2	35.6	28.8	42.7	36.6	29.8	43.8
Unknown	54	79.6	66.5	89.4	48.1	34.3	62.2	42.6	29.2	56.8

^a^Participants reported their earnings in Tanzanian shillings in terms of either daily, weekly or monthly income. Reported daily and weekly earnings were converted to monthly earnings on the assumption that each participant worked for 22 days per month. The conversion to US Dollars (USD) was based on the exchange rate at the time of data collection (1USD = 1,887 Tanzanian shillings). ^b^A higher quintile indicates higher socio-economic status. The socio-economic status indicator was derived as a latent variable from 19 indicators collected in the questionnaire including education, earnings and household ownership of car, fridge, television and motorcycle

### Associations with symptoms of poor mental health

Overall, about 40% (95% CI: 37–43%) of women reported symptoms of poor mental health based on SRQ-20 cut-off point of 7/8. In Table 5, we present the associations between experience of IPV and abuse during the past 12 months and symptoms of poor mental health. In general, the proportion of women with symptoms of poor mental health was relatively higher among women experiencing IPV and abuse. After we adjusted for age and socio-economic status, women who experienced physical violence (OR = 1.8; 95% CI: 1.3–2.7) and sexual violence (OR = 2.8; 95% CI: 1.9–4.1) were significantly more likely to report symptoms of poor mental health when compared to other women. Similarly, women reporting to have experienced emotional abuse (OR = 1.5; 95% CI: 1.1–2.2), and economic abuse (OR = 1.9; 95% CI: 1.4–2.6) were more likely to report symptoms of poor mental health after we adjusted for age, socio-economic status and experience of violence.

**Table 5:** The associations between experience of intimate partner violence and abuse during the past 12 months and symptoms of poor mental health among ever-partnered women in Mwanza, Tanzania

	*N*	Poor mental health status *n* (%)	Odds ratio	95% CI		*P*	Adjusted odds ratio (AOR)^a^	95% CI		*P*
Physical violence										
Experienced in past 12 months	194	113 (58.2)	2.40	1.74	3.32	<0.001	1.84	1.27	2.65	0.001
No Experience in past 12 months	827	299 (36.2)	1				1			
Sexual violence										
Experienced in past 12 months	171	110 (64.3)	3.34	2.34	4.78	<0.001	2.76	1.86	4.09	<0.001
No Experience in past 12 months	850	302 (35.5)	1				1			
Controlling behaviour										
Experienced in past 12 months	639	277 (43.3)	1.37	1.05	1.78	0.022	0.97	0.71	1.31	0.832
No Experience in past 12 months	382	135 (35.3)	1				1			
Economic abuse										
Experienced in past 12 months	343	194 (56.6)	2.71	2.06	3.57	<0.001	1.86	1.35	2.57	<0.001
No Experience in past 12 months	678	218 (32.2)	1				1			
Emotional abuse										
Experienced in past 12 months	401	213 (53.1)	2.40	1.84	3.13	<0.001	1.53	1.09	2.15	0.015
No Experience in past 12 months	620	199 (32.1)	1				1			

^a^Adjusted odds ratio estimates were obtained from the analysis restricted to 967 participants with complete data on socio-economic status. Each model for violence was adjusted for age, socio-economic status, and other violence. Each model for abuse was adjusted for age, socio-economic status, violence and other abuses

## Discussion

In this paper, we present baseline results from a study of women attending microfinance loan groups enrolled in the ongoing MAISHA study in Mwanza, Tanzania. We found a high prevalence of IPV, with 61% of women reporting ever experiencing physical and/or sexual IPV and 27% of women reporting it in the past 12 months. A substantial proportion of women reported severe physical violence which happened relatively frequently, suggesting that this is a common experience among women in this population. We also found a high prevalence of abuses, confirming that both violence and abuse from an intimate partner are common social, public health and human rights concerns among women in this study population.

The prevalence of sexual and/or physical violence in this population is consistent with previous studies conducted in Tanzania [3, 8, 18] and in other countries in sub-Saharan Africa [1]. To our knowledge, this is one of the first studies to examine the prevalence of emotional and economic abuse and controlling behaviour in a relationship in an African setting. A substantial proportion of women reported economic and emotional abuse in the last 12 months and almost two thirds of women reported experiencing at least one form of controlling behaviour during the same time period, suggesting this may constitute a form of normal behaviour in this Tanzanian population. We observed high rates of co-occurrence of reported IPV and abuses, with almost all women who reported experiencing physical and/or sexual violence in the last 12 months also reporting to have experienced one or more forms of abuse during the same time period. This high degree of overlap suggests that women in this population are more likely to experience both violence and multiple forms of abuse from their intimate partners. While more in-depth research is required to understand the impact of the different forms of abuse and controlling behaviour, our findings suggests that comprehensive violence prevention intervention programmes areneededto address IPV and more neglected forms of abuses [20].

We examined the associations between the prevalence of IPV and abuses in the last 12 months and socio-demographic characteristics. In general, IPV (and to some extent controlling behaviour) varied by socio-demographic characteristics, with much higher prevalence rates among younger women, women with young partners and women with less education. This is also in line with prior studies reporting women below the age of 25 having higher rates of violence than women aged 25 or older [21]. Potential reasons for this association are that relationships in younger age groups are more likely to be less stable, and to be associated with early pregnancy and higher levels of financial stress [22]. It is also possible that the levels of recall and social desirability bias is relatively lower among young women and therefore the likelihood of reporting IPV is higher in this population [23]. We observed relatively lower prevalence of IPV and abuses among women who were widowed and those without children below the age of 18 years. This is likely to be related to other factors, such as age, that are related to IPV in this population. Surprisingly, women’s monthly earnings and socio-economic status were not found to be related to experiences of violence or abuses. This finding warrants further investigation in order to examine the role of socio-economic status as a risk factor for IPV and abuses.

We found that women who reported experiencing IPV during the past 12 months were more likely to report symptoms of poor mental health, and this association remained unchanged after we adjusted for age and socio-economic status. This is consistent with findings from other studies [5, 24, 25]. We also found that economic and emotional abuses were significantly associated with symptoms of poor mental health. These cross-sectional findings suggest a strong association between IPV/abuses and symptoms of poor mental health, although it is not possible to confirm a causal link between them. Women who report symptoms of poor mental health may be more likely to experience violence [5], suggesting that poor mental health may be a risk factor for IPV. It is also possible that experience of severe and prolonged IPV may lead to poor mental health and other adverse health outcomes, as previously reported in prospective studies [26–28].

The high prevalence of IPV and abuses and its strong links with symptoms of poor mental health underline the importance of developing and testing appropriate interventions. As part of the MAISHA study, we are conducting a cluster randomised controlled trial to assess the impact of microfinance combined with gender training for women on participants’ experience of physical and/or sexual IPV, as well as other gender-empowerment, economic, and health related outcomes in Mwanza, Tanzania. We have enrolled 1049 women receiving group-based microfinance loans and successfully delivered 10 sessions of gender training to women randomised to the intervention arm of the trial. The impact of the intervention will be assessed through a face-to-face interview during a follow-up survey to be conducted in 2017, approximately 29–30 months after randomisation.

The findings of this cross-sectional survey need to be considered in view of several limitations. Firstly, reporting of IPV may be prone to under-reporting due to social desirability or shame as women are commonly stigmatized and blamed for the abuse they receive. Several measures were implemented to minimise this problem, including training of interviewers to adhere to high ethical standards, and closely following the WHO guidelines on researching violence against women [29]. Secondly, the study was conducted among a sample of women receiving microfinance loans living in Mwanza, Tanzania’s second largest city. It may therefore not be representative of the whole of Tanzania. However, this does not affect the validity of the findings presented, especially since the prevalence rates are comparable to previous studies. Thirdly, we analysed cross-sectional data collected at baseline, and this does not allow any inferences to be made regarding causality and temporality with respect to the socio-demographic factors and health outcomes assessed. Lastly, women’s mental health status was assessed through self-report of symptoms and does not represent a clinical diagnosis.

## Conclusions

In conclusion, these findings show that violence by an intimate partner is a major problem among women in Tanzania. We also found high levels of economic and emotional abuse and very high levels of controlling behaviour, with high rates of co-occurrence of reported IPV and abuses. The prevalence of IPV and abuses varied by socio-demographic characteristics, showing much higher prevalence rates among younger women, women with young partners and less educated women. The high prevalence of IPV and abuses and its strong links with symptoms of poor mental health underline the urgent need for developing and testing appropriate interventions in settings like Tanzania to tackle both violence and abusive behaviours among intimate partners. As part of the ongoing MAISHA study, we aim to assess the impact of microfinance combined with gender training for women on physical and/or sexual IPV in NW Tanzania.
